# Fatal pulmonary embolism following splenectomy in a patient with Evan’s syndrome: case report and review of the literature

**DOI:** 10.1186/s12959-017-0141-5

**Published:** 2017-07-03

**Authors:** Varun Monga, Seth M. Maliske, Usha Perepu

**Affiliations:** 1 Division of Hematology, Oncology, and Blood and Marrow Transplantation, University of Iowa Carver College of Medicine, Iowa City, IA 52242 USA; 2 Aspirus Wausau Hospital Regional Cancer Center, 333 Pine Ridge Blvd, Wausau, WI 54401 USA

**Keywords:** Venous thromboembolism, Splenectomy, Evan’s Syndrome, Reactive thrombocytosis, Case report

## Abstract

**Background:**

Evans syndrome (ES) is a rare disease characterized by simultaneous or sequential development of autoimmune hemolytic anemia (AIHA) and immune thrombocytopenia (ITP) with or without immune neutropenia. Splenectomy is one of the treatment options for disease refractory to medical therapy. Venous thromboembolism (VTE) following splenectomy for hematological diseases has an incidence of 10%.

**Case presentation:**

Here we describe a case report of a young patient hospitalized with severe hemolytic anemia with Hgb 4.8 g/dl. He developed thrombocytopenia with platelet nadir of 52,000/mm^3^, thus formally diagnosed with ES. He failed standard medical therapy. He underwent splenectomy and had a fatal outcome. Autopsy confirmed the cause of death as pulmonary embolism (PE).

**Conclusions:**

This case report and review of the literature highlight important aspects of the association between VTE, splenectomy, and hemolytic syndromes including the presence of thrombocytopenia. The burden of the disease is reviewed as well as various pathophysiologic mechanisms contributing to thromboembolic events in these patients and current perioperative prophylactic anticoagulation strategies. Despite an advancing body of literature increasing awareness of VTE following splenectomy, morbidity and mortality remains high. Identifying high risk individuals for thromboembolic complications from splenectomy remains a challenge. There are no consensus guidelines for proper perioperative and post-operative anti-coagulation. We encourage future research to determine which factors might be playing a role in increasing the risk for VTE in real time with hope of forming a consensus to guide management.

## Background

Evans syndrome (ES) is a rare disorder first described by Evans et al. [1] in 1951. Primary ES comprises a combination (either simultaneous or sequential) of: 1) autoimmune hemolytic anemia (AIHA) characterized by hemoglobin less than 11 g/dl, low haptoglobin level, elevated lactate dehydrogenase (LDH) and/or bilirubin levels, and positive direct antiglobulin test, and 2) immune thrombocytopenia (ITP) defined as platelet count below 100 × 10^9^/L on 2 separate occasions, with or without 3) immune neutropenia (neutrophil count below 1.5 × 10^9^/L on 2 separate occasions at least a week apart), in the absence of an underlying etiology. Secondary ES may be associated with other conditions such as systemic lupus erythematosus, lymphoproliferative disorders, or primary immunodeficiencies. The incidence of primary ES is reported as 0.8% to 3.7% in patients with either ITP or AIHA [2]. There is no gender predilection. Due to the lack of randomized or prospective trials, the management of ES remains anecdotal. Here we describe a case of a young male patient diagnosed to have ES who died of a pulmonary embolism following splenectomy.

## Case presentation

The patient was a 36-year-old man with a past medical history significant for alcoholism and morbid obesity (body mass index (BMI) 41.9 kg/m2). He presented to an outside hospital with progressive weakness, dyspnea, and dark urine. Laboratory values showed profound anemia with hemoglobin of 4.8 g/dL, hematocrit 14.9% (mean corpuscular volume (MCV) not available), total bilirubin of 6.1 mg/dL, LDH 400 U/L, and haptoglobin <20 mg/dL which is suggestive of hemolysis. Platelet count was 219,000/mm^3^. Electrocardiogram (EKG) showed signs of subendocardial ischemia. He was transferred to the University of Iowa Hospital for further evaluation. On examination he was pale, tachycardic, diaphoretic, jaundiced, and without organomegaly on palpation due to his body habitus. Repeat hemoglobin on arrival was 3.5 g/dl with an MCV of 90 fl. The peripheral smear showed marked normochromic normocytic anemia, a normal reticulocyte count of 28 k/mm^3^, slight lymphopenia, and toxic neutrophils. There was no morphologic evidence of intravascular or extravascular hemolysis, and INR, PTT, Fibrinogen, and D-Dimer were normal, signifying absence of disseminated intravascular coagulation. Erythropoietin level was high at 1420 mU/ml with a normal serum creatinine of 1.0 mg/dl. The direct coombs test was positive with a low affinity IgG warm autoantibody, IgG positivity and complement positivity. Paroxysmal nocturnal hemoglobinuria was ruled out by flow cytometry. Ultrasound of the abdomen showed moderate splenomegaly with spleen measuring 19.2 × 8.3 × 7.7 cm.

**Fig. 1:**
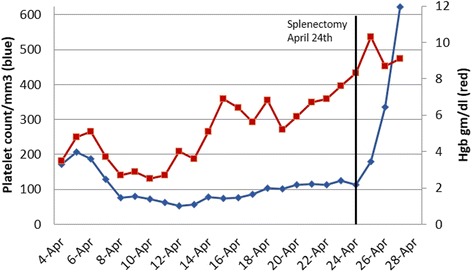
Trend of patient’s platelet count (*blue line*) and hemoglobin (*red line*) over the hospital stay with respect to timing of splenectomy. Follow-up platelet count and hemoglobin on May 1 (not depicted on the graph) was 1,204,000/mm^3^ and 10.6 g/dl, respectively

He was started on oral prednisone at 1 mg/kg daily and folate for AIHA. Due to ongoing anemia with hemoglobin 4.8 g/dl, he was given a daily dose of intravenous immunoglobulin (IVIG) on days 2, 3, and 4. On Day 4 of admission the platelet count dropped to 76,000/mm3 in absence of any heparin products or other etiology for thrombocytopenia prompting a diagnosis of *Evans syndrome*. By day 4, he had received 12 units of packed red blood cells. Despite these efforts, his anemia persisted, and even worsened, as hemoglobin decreased to 2.6 g/dl. He developed chest pain due to severe anemia. On hospital day 5, Rituximab (375 mg/m2) and Cyclophosphamide (500 mg/m2) was initiated for persistent, worsening severe anemia. A bone marrow biopsy was performed for severe unresponsive anemia. It showed hypocellular bone marrow (20–40%) with erythroid hyperplasia, decreased granulopoiesis, increased megakaryocytes, no increase in immature cells, normal conventional cytogenetics, and normal fluorescent in situ hybridization studies. The cell counts eventually began to rise with transfusion support as hemoglobin trended up to 6.8 g/dl with 4 additional units of blood over the next 5 days. He was re-dosed with two doses of Rituximab on days 11 and 18 as well as one dose of Cyclophosphamide on day 12. After nearly 3 weeks in the hospital, the hemoglobin had not risen above 6.9 g/dl (Fig. 1) prompting a discussion for splenectomy. He was appropriately vaccinated prior to surgery. He, also, developed a catheter associated blood stream infection with methicillin sensitive *Staphylococcus aureus* which was treated with intravenous antibiotics. He underwent laparoscopic splenectomy on Day 20 of admission. The procedure was uncomplicated with operative time of 90 min and minimal blood loss reported as less than 50 ml. Pathology of the spleen revealed splenomegaly of 950 g with extramedullary hematopoiesis and multiple areas of necrosis.

**Fig. 2:**
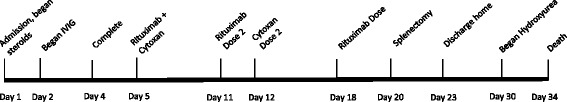
Timeline of treatment

Of note, during the patient’s protracted hospital stay, prophylactic pharmacologic anticoagulation was withheld until splenectomy due to ongoing thrombocytopenia with risks for bleeding. Platelet nadir was 52,000/mm^3^. In accordance with American College of Chest Physicians (ACCP) antithrombotic guidelines [3] sequential compression stockings (SCDs) were initiated on Day 3 of admission. Following splenectomy, the patient had a rise in his platelet count (Fig. 1) above 100,000/ mm^3^ and at that time was started on subcutaneous enoxaparin 40 mg/day for deep venous thrombosis (DVT) prophylaxis. The patient underwent physical therapy and was discharged home on Day 23 with a hemoglobin level of 9.6 g/dl and platelet count of 623,000/mm^3^. No extended prophylaxis was provided upon discharge. He was followed in the clinic a week later where thrombocytosis was identified with a platelet count of 1,204,000/ mm^3^ (normal range 150,000–400,000/ mm^3^). Hydroxyurea commenced at a dose of 500 mg/day and he was given a dose of Rituximab. Four days later the patient was found unresponsive at home and resuscitative efforts at the local emergency room failed. An autopsy revealed bilateral massive pulmonary embolism (PE). There was no liver cirrhosis and no portal or splenic vein thrombosis (PSVT). The anterior, lateral, and posterior aspects of the left ventricle and the interventricular septum of the heart showed healing areas of myocardial infarction attributed to the severe anemia during the previous hospitalization (Fig. 2).

## Discussion

This case highlights the risks of venous thromboembolism (VTE) affiliated with splenectomy in a hemolytic syndrome. Our patient had a fatal outcome related to VTE in the post-operative setting. Initiatives by such organizations as the National Quality Forum and the Joint Commission have aimed to improve recognition of the risk of VTE in hospitalized patients [4, 5]. In fact, to highlight the importance of venous thromboembolism in this setting, the Centers for Medicaid and Medicare (CMS) has deemed it a “never-event”, when specifically following orthopedic procedures [6].

Pharmacologic prophylaxis with subcutaneous Lovenox, Heparin, or Fondaparinux was withheld in this patient because the risk of bleeding with thrombocytopenia and a severe hemolytic syndrome with profound anemia seemed to outweigh the benefit of pharmacologic prophylaxis. Therefore, sequential compression devices were used in place of pharmacologic agents. However, the risk of venous thromboembolism was likely underestimated in this patient. He had several inherent risks for VTE including morbid obesity, prolonged hospital stay, underlying hemolysis, central venous line access throughout much of the hospitalization, sepsis, and major surgery.

Prophylactic Lovenox was held until his platelet count rose above 100,000/mm^3^. Guidelines suggest withholding any dose of anticoagulation if the platelet count is less than 50,000/mm^3^; however, for very high-risk patients, the continued use of prophylactic anticoagulation therapy can be considered if the platelet count is more than 30,000/mm^3^ [3]. However, thrombocytopenia does not always correlate with risk of thrombosis [7]. Hence, diligent use of anticoagulation is even more important in patients with thrombocytopenia where the decision for or against pharmacologic prophylaxis should be made on a case-by-case basis.

Expert consensus panels currently do not address the use of prophylactic anticoagulation in the settings of post-splenectomy for hematological disease and/or reactive thrombocytosis [8–14]. Without guidelines or randomized trials directing evidence-based use of anticoagulation, we must rely on case series and case studies. The goal of the subsequent discussion is to investigate burden of disease, mechanism of risk, and best treatment modalities in hopes of better understanding risk for VTE in these patients and help encourage dedicated therapies to prevent similar outcomes.

### Burden of disease

Splenectomy is utilized in the diagnosis and treatment of a variety of hematologic disorders unresponsive to medical management in hopes of reducing the frequency of relapse and lowering the maintenance dose of steroids. Splenectomy, when performed for hematologic disorders, is associated with a complication rate as high as 40% [15]. Complications include perioperative bleeding, overwhelming infection with encapsulated organisms and a growing body of evidence is showing a heightened risk of peri-operative and post-operative venous and arterial vascular events, especially venous thromboembolism. Splenectomy is a known risk factor for VTE independent of the underlying indication (i.e.: malignancy) or comorbidities (i.e.: antiphospholipid syndrome) [16, 17]. It has an associated 5-fold increased risk of fatal PE [18]. In addition, warm autoimmune hemolytic anemia (w-AIHA) is associated with a heightened risk of VTE with an incidence of 15–33% [19–22]. Risk appears to be higher in patients with a more severe hemolytic process highlighted by lower hemoglobin levels and marked hemolysis with higher reticulocyte counts, bilirubin and LDH levels, and lower haptoglobin levels. In these studies, splenectomy did not appear to increase the risk of VTE relative to the baseline risk associated with w-AIHA [22, 23]. The incidence of thromboembolic complications following splenectomy when specifically performed for hematological diseases has been reported to range from 10 to 30% which includes PSVT, PE, and DVT [17, 24]. Despite these reports, VTE risk following splenectomy in the background of w-AIHA, or related hemolytic processes, has not been reported in depth in the literature.

In the original case series of patients having undergone splenectomy for ES, AIHA, or ITP, there was no report of VTE [1]. In another case series of 12 patients with ES, one patient died of pulmonary embolism 6 months after diagnosis, however this patient did not undergo splenectomy. Three of the 12 underwent splenectomy, none of whom had reported post-procedural venous thromboembolic events [2]. In similar cases with AIHA and ITP, there were no cases of post-splenectomy VTE [25, 26]. However, evidence for the association of VTE with splenectomy unfolded over ensuing decades. In a case series of patients with hemolytic anemia, 13% of patients developed thromboembolic complications following splenectomy, and two patients died because of VTE [27]. In one large population based study looking at incidence of thromboembolism in post-splenectomy ITP the procedure was associated with a 4.3% higher cumulative risk of VTE [28]. A Dutch study reviewing 563 splenectomies, identified 45 cases performed for hemolytic anemia, of which, 9% (4 out of 45 patients) acquired portal vein thrombosis [29]. A retrospective chart review reported 3 out of the 6 patients who underwent laparoscopic splenectomy for AIHA developed thrombosis in the portal venous network. Each patient also had post-splenectomy thrombocytosis with platelet counts ranging from 500 K/mm^3^ to 896 K/mm^3^. One of these patients also developed a concomitant DVT. None of them had received prophylactic anticoagulation during the perioperative period [30]. Mohren et al. [31] highlight events in a case similar to ours in which a 24-year old female patient with AIHA who underwent splenectomy for continued anemia despite medical therapy. She received appropriate prophylactic dose anticoagulation during her hospital say. After discharge, she developed extensive pulmonary embolism, portal vein thrombosis, and mesenteric vein thrombosis. She subsequently died due to bowel ischemia. The platelet count reported at the time of death was 499,000/L.

Reit [29] and Winslow [32] have individually demonstrated that hemolytic anemia is an independent risk factor for PSVT-- independent of splenectomy and thrombocytosis. Systemic venous thrombosis has also been observed in patients with hemolytic syndromes who have undergone splenectomy. In a case series of 47 patients with AIHA (6 of whom had ES), venous thromboembolism was the most common cause of death. Each patient who had succumbed to VTE had undergone splenectomy during their treatment and was on corticosteroid therapy at the time of death. None of the cases of Evan’s Syndrome was associated with a thrombotic complication [33]. The risk for thrombosis extends beyond immune-mediated hemolytic processes. In a large case-series looking at Thalassemia intermedia patients, this hemolytic process was associated with an increased incidence of venous thromboembolism beyond the portal circulation (32% DVT, 16% PSVT and 13% PE) [34]. In addition, there have been numerous case reports of thrombosis after splenectomy in patients with hereditary stomatocytosis, a hemolytic syndrome characterized by anemia secondary to abnormalities in red cell cation permeability. In fact, post-operative thrombosis is so prevalent that splenectomy is no longer recommended in the treatment of hereditary stomatocytosis [35].

The burden of disease is further complicated by the risk for post-splenectomy reactive thrombocytosis (RT). This phenomenon is common especially in patients with splenomegaly, occurring in 75% of cases [36], and is characterized by a mild asymptomatic thrombocytosis reaching a peak at the end of the 2nd week and subsiding by 3 months post-splenectomy [26]. Typically, RT is not considered a risk factor for thromboembolic complications, as only 1.6% of patients had thrombotic event in only large case-series, with a majority occurring in setting of other significant risk factors including post-surgery and malignancy [37]. Therefore, reactive thrombocytosis following splenectomy carries a more modest risk. One study identified a 5% risk of PSVT [38]. Even in cases of extreme reactive thrombocytosis (platelet count >1,000,000/mm^3^), the risk of thrombotic complication is relatively modest (4–5%) [39]. In fact, increasing platelet count has inconsistently shown a correlation with risk of thrombosis [40]. What seems to be a greater driver of post-splenectomy thrombotic risk is the evidence of ongoing hemolysis. When splenectomy is effective at ameliorating hemolysis, thrombocytosis is usually milder and the risk of thrombosis is low. However, with ongoing hemolysis, thrombocytosis may persist, and is associated with a higher thrombotic potential [27] (Table 1).

**Table 1:** Case studies showing the type of thrombosis in patients undergoing splenectomy for hematological disorders

Study	Hematological disease	Type of thrombosis	Secondary thrombocytosis	Median interval to thrombosis	Patient outcome
Evans et al. [1]	12 ES	None	Present in 2 out of 12	N/A	N/A
NG et al. [2]	3 ES	None	Unknown	N/A	N/A
Silverstein et al. [25]	4 ES	Unknown	Unknown	Immediate post op	2 patients died
Michel et al. [26]	19 ES	None	Unknown	N/A	N/A
Boyle et al. [28]	1762 ITP	27 PSVT 75 VTE	Unknown	PSVT – 9.3 mo VTE – 20.9 mo	PSVT – 5 yr. survival 38% VTE – 5 yr. survival 62%
Van riet et al. [29]	4 HA	PSVT	Present	12 days	Unknown
Mohren et al. [31]	1 AIHA 1 ITP	PE & PSVT PE & PSVT	Present Present	8 days 7 days	Died Survived
Winslow et al. [32]	3 HA	PVT	Present	11 days	Survived
Allgood et al. [33]	47 AIHA		Unknown	Unknown	
Stamou et al. [38]	1 AIHA	PSVT	Present	7 days	Survived
Tiede et al. [69]	1 ITP	PE	Unknown	30 days	Survived
Fujita et al. [41]	2 AIHA	PSVT	Present	16 days	Survived

*ES* Evan’s Syndrome, *ITP* Immune-mediate thrombocytopenic purpura, *HA* hemolytic anemia, *AIHA* autoimmune-mediate hemolytic anemia, *PSVT* portal splenic venous thrombosis, *PE* pulmonary embolus, *DVT* deep vein thrombosis, *mo* months, *N/A* not applicable

### Pathophysiology of venous thrombosis post splenectomy – Local vs. systemic factors

Proposed mechanisms of thrombosis are vast and include local and systemic factors. These local and systemic factors contribute to all thrombosis, portal/splenic vein thrombosis, deep vein thrombosis, and pulmonary embolus, alike. Local factors and systemic factors that promote thrombosis are centered upon Virchow’s triad. Local factors center around hemostasis, while systemic influences include, but are not limited to, platelet abnormalities and the post-operative inflammatory state which leads to hypercoagulability, endothelium injury, and platelet activation.

Stasis of blood in the splenic vein remnant appears to be a primary causative factor for PSVT [29, 41]. Splenomegaly is a risk factor for stasis of blood. This has been attributed to the relationship of spleen mass to size of splenic vein stump. Increasing size of the splenic vein stump promotes venous stasis which enhances the risk for thrombosis [42]. Danno et al. [43] confirmed that a pre-operative splenic vein diameter greater than 8 mm was an independent predictor of PSVT. Similarly, the higher incidence of PSVT noted with removal of heavier spleens have been attributed to longer surgical time and prolonged increased intraabdominal pressure. Prolonged exposure to increased intraperitoneal pressures during laparoscopic procedures promotes venous stasis within the portal venous network by decreasing portal blood flow [44]. A recent prospective study identified a 55% higher incidence of PSVT noted with laparoscopic compared to open splenectomy [45]. This contradicts reports by Winslow [32] (5% in laparoscopic vs 9% in open), Chaffanjon [42] (6.7% with open), and Valeri [46] (8.3% with open), and was not seen in the animal study done by Lyass [47]. It is likely that these retrospective studies are biased based on patient selection and that the etiology of thrombosis is likely multifactorial.

The systemic mechanisms that may promote VTE in splenectomized patients are unclear, but a vast number of systemic influences have been proposed to promote thrombosis, including endothelial activating factors, increased platelet activation and/or released microparticles [35, 48]. Further pro-thrombotic mechanisms have been proposed, primarily centered on the continued intravascular hemolysis despite splenectomy. The spleen primarily functions in filtering and removing unwanted bloodstream products which can include infectious organisms, cellular debris, senescent cells, or immunologic-marked cells. In the absence of this inherent phagocytic property, ongoing hemolysis allows thrombogenic microparticles formed from erythrocyte fragmentation to remain in the bloodstream. Hemolysis in the absence of splenic sequestration also promotes hyperviscosity secondary to retained intracellular inclusions (i.e.: hemolytic byproducts). Persistent hemolysis also drives activation of vascular endothelium which increases platelet adherence. Lastly, ongoing hemolysis is a stimulus for thrombopoiesis. When thrombopoiesis exceeds the potential for platelet removal, thrombocytosis results. Independent of hemolysis, persistence of abnormal erythrocytes in circulation is also postulated to contribute to thrombosis [35]. Further, adhesive platelets’ pre-hemolytic thromboplastic activity and significant production of anti-plasmin as a result of splenectomy are additional proposed factors contributing to post-splenectomy thromboembolism [17].

Qualitative and quantitative platelet abnormalities also promote a thrombogenic potential. Gordon et al. [49] described post-splenectomy PSVT associated with these platelet abnormalities in the setting of myeloproliferative and hemolytic disorders. These platelet anomalies can originate in several fashions. First, splenectomy, by itself, can precipitate a rise is inflammatory cytokines which, in turn, mediates a hypercoagulable state. In addition, the absence of the spleen gives rise to high levels of markers of persistent platelet activation, namely platelet microparticles (PMP) and CD26P [41]. This promotes thrombosis independent of the platelet count. As discussed previously, there is mixed evidence in support of relationship between higher platelet counts and risk for thrombosis [40]. Despite this, it is most evident that a majority of thrombotic events occur with platelet counts >600,000/mm^3^ [50]. The exact mechanism(s) that drives clotting vs. bleeding associated with thrombocytosis has not been elucidated.

Reticulated platelets (RP) are the high ribonucleic acid (RNA) containing young platelets in circulation and are an index of thrombopoiesis. Reticulated platelet percentage (RP%) and absolute RP count have been shown in a study [51] to be significantly increased in the state of transient thrombocytosis, including the post-splenectomy setting. Moreover, higher RP% and absolute RP count had a positive predictive value of 38% and 88%, respectively, for developing symptomatic thrombosis. This study was unable to show a causal relation of secondary thrombocytosis as a prothrombotic state. A similar study by Ryningen et al. [52] confirmed the above finding and also showed a significantly elevated RP% in immune thrombocytopenic purpura. This was also proposed in the surgical literature [29, 32]. Considering the above studies, patients with secondary Evans syndrome associated with an underlying autoimmune phenomenon who undergo splenectomy are undoubtedly at a profound risk for developing thrombotic complications. RP% and absolute RP count if validated in prospective trials may help to guide therapy in patients who are at higher risk of developing thrombosis.

Lastly, high dose oral steroid treatment, which forms the first line treatment of ES, is increasingly being recognized as a prominent risk factor in producing a prothrombotic state [53, 54]. Stuijver et al. [55] demonstrated the risk of PE was highest in the first 30 days of glucocorticoid exposure and is associated with a 10-fold increased risk in patients receiving higher doses of steroids, prednisolone >30 mg, or its equivalent.

### Management of perioperative thrombotic risk

Due to lack of sufficient data in patients with Evans syndrome, most of the treatment strategies are extended from studies done in essential thrombocythemia (ET), ITP, and various chronic autoimmune hemolytic anemia syndromes. Corticosteroids are considered the first line therapy which our patient received. IVIG and Rituximab are found to be especially useful in patients with refractory ITP or chronic severe steroid dependency. In our patient, each of the above treatment modalities were implemented. Given the lack of response, splenectomy was rightfully chosen as the next best step. Knowing splenectomy and steroids both carry a heightened risk for thrombotic events, it is important to always consider both pharmacologic and mechanical DVT prophylaxis.

Low dose aspirin therapy, 81 mg, is standard of care in low to moderate risk ET, defined as presence of JAK2 mutation or cardiovascular disease but age < 60, platelet count less than 1,500,000/L, and no history of thrombosis. Aspirin use has been associated with improved outcomes in the setting of secondary causes of thrombocytosis [39]. Therefore, many are advocating for standard use of aspirin in those with RT. Aspirin is indicated as a standard therapy in the comorbid cardiovascular conditions regardless of RT but in the presence of RT especially in older patients, it may be protective. This lends evidence that aspirin use for RT in the presence of other thrombotic risk factors may prove to be beneficial in elderly patients but there is still no reliable approach to predict who would benefit from aspirin.

In higher risk ET, aspirin is used with a cytoreductive agent such as hydroxyurea or anagrelide. As per a large randomized study [56] performed in patients with ET who were at high risk for thrombosis, anagrelide plus aspirin had a lower rate of venous thrombosis but a higher rate of arterial thrombosis as compared to hydroxyurea plus aspirin. As the incidence of venous thrombosis in untreated patients with high risk ET is unknown, it was unclear if this rate was increased by hydroxyurea. It is well known that the incidence of arterial thrombosis is more than three times the venous thrombosis in ET. The same is unfortunately not well established in case of RT and it is difficult to extrapolate this data to our patient with RT. Granted, in our patient, hydroxyurea was used as a cytoreductive therapy and theoretically could have contributed to venous thrombosis. More data needs to be collected to better elucidate the risks of hydroxyurea vs anagrelide in regards to thrombosis.

Melphalan has been used in hemorrhagic thrombocythemia post-splenectomy as a cytoreductive agent and was proven to be effective in controlling the platelet proliferation thereby reducing the bleeding complications in some situations [57]. The role of Melphalan has not been investigated in depth over the ensuing decades and could be studied further in patients with RT to prevent thrombotic events and/or be used as a more effective agent compared to hydroxyurea or anagrelide.

Plateletpheresis has been shown to be a useful method to rapidly decrease the platelet counts especially in patients with acute thromboembolic events of neurologic or pulmonary origin [58]. Perioperative plateletpheresis was used during microsurgical free tissue transfer in a patient with post-splenectomy thrombocytosis [59]. One of the largest reviews reported more symptomatic events in patients with platelet counts greater than 1,500,000/mm^3^ and treatment benefit reached statistical significance in patients with ET but not in patients with post splenectomy reactive thrombocytosis. Another small case series [60] of five patients with underlying myeloproliferative neoplasm (MPN) and complications of thrombocytosis reported beneficial effects of plateletpheresis. Most patients required at least 2 to 3 procedures for control of platelet counts. This procedure does come with its own risks of bleeding, infection, arrhythmias, hypotension, and cost. There is lack of controlled trials to make any recommendation for or against this procedure especially in patients with RT. As discussed previously, the existing literature is inconsistent in its association between platelet count and thrombosis. Plateletpheresis should therefore not be used as a prophylactic measure. Its use in acute events is questionable and shouldn’t be used in the first line setting. However, its use can be considered in acute, symptomatic, uncontrolled events refractory to other cytoreductive agents.

A great unanswered question in regards to treatment is how best to prevent thrombosis. As discussed in previous sections the inherent risks factors for thrombosis following splenectomy are extensive. Our patient had several inherent risks for VTE including morbid obesity, prolonged hospitalization with bed rest, underlying hemolysis, sepsis, and major surgery. He also had central venous access for much of the hospital stay. The 2005 version of the Caprini Risk Assessment Model [61] is the most widely used and well-validated risk prediction for postsurgical patients and is referenced in the ACCP guidelines [3]. The updated version of this model stratifies risk into five categories- lowest, low, moderate, high, and highest. Our patient had a Caprini VTE Risk Assessment score of 8 which carries a 6.0% risk of VTE without anticoagulation. With this risk, the Caprini model advocates for out of hospital use of anticoagulation for at least 7–10 days [62]. In addition, the incidence of thromboembolic complications following splenectomy when specifically performed for hematological diseases, as previously mentioned, has been reported to range from 10 to 30% [17, 24]. With this added risk, Caprini would advocate for 30 days of anticoagulation.

Prophylactic anticoagulation is used outside the hospital in various settings including after orthopedic surgery [63], following major abdominal surgery in setting of cancer [64], following bariatric surgery [65], and after spinal cord injuries [66]. With the inherent risks associated with splenectomy for hematological diseases, and a high Caprini VTE risk score, our patient may have benefited from outpatient anticoagulation. Prophylactic subcutaneous heparin alone is known to be insufficient to prevent the development of PSVT [32]. Prior recommendations have included combination therapy with antiplatelet agents, warfarin, and heparin. Our patient’s BMI exceeded 40 kg/m^2^. Caution must be exercised when offering prophylactic anticoagulation for patients with extreme BMI’s as high and low BMI’s are not well-represented in clinical trials. Use of Lovenox 40-60 mg twice daily for 30 days may have prevented our patient’s fatal outcome [67].

Unpredictable use of pharmacologic prophylaxis stems from fear of bleeding. Bleeding risk associated with RT is not well-described in the literature. It is important to note that the platelets in RT are normal and therefore it carries a theoretical lower risk for bleeding compared to ET, as in ET, platelets are abnormal. An acquired Von Willebrand factor deficiency has also been described in ET. This is associated with increased risk of bleeding [58]. Again, this is not reported in patients with RT. Because of the known risks of bleeding with ET, many of the same fears are carried over to RT. This has likely contributed to the inconsistent use of anticoagulation in these patients. In addition, associated pre-operative thrombocytopenia and severe anemia, as seen in our case, offers further reluctance to use anticoagulation. However, thrombocytopenia does not always correlate with risk of thrombosis. In fact, the rate of VTE in hematologic malignancies, where platelet counts are often profoundly low, mirrors the frequency seen in solid tumors [7]. This has prompted one group to suggest decreasing dose of LMWH by 50% when platelet counts are below 50,000/mm^3^, as opposed to eliminating pharmacologic prophylaxis altogether in that setting [68]. Hence, diligent use of anticoagulation is of utmost important in patients with thrombocytopenia where the decision for or against pharmacologic prophylaxis should be made on a case-by-case basis.

Proper use of prophylactic dose anticoagulation needs to become a greater priority in these patients. However, prophylactic decisions like these remains difficult without consensus guidelines. Expert consensus panels currently do not address the use of prophylactic anticoagulation in the settings of post-splenectomy for hematological disease and/or reactive thrombocytosis [8–14]. Without guidelines or randomized trials directing evidence-based use of anticoagulation, we must rely on case series and case studies. We encourage further efforts to investigate optimal length of therapy. It is possible anticoagulation needs to be extended several weeks beyond the hospitalization as directed by the Caprini risk assessment. Another proposal is to use anticoagulation until there is no further evidence of hemolysis which would require close follow-up with dedicated laboratory surveillance.

## Conclusion

Hemolytic Syndromes such as Evan’s Syndrome carry an inherent risk for VTE and those treated with steroids and or splenectomy are exposed to a greater burden of risk for thrombosis. The mechanism behind this thrombotic potential has been discussed in the literature, but the risks remain poorly understood across the medical community. Management of Evans syndrome especially in those who are refractory to medical therapy remains difficult. Splenectomy was and currently is a good second line treatment option. However, venous thromboembolism has become an emerging post-operative complication. The variability of platelet counts and the incidence of thrombotic events post-splenectomy pose a significant challenge to determine which patients need to be treated with thromboprophylaxis and or cytoreductive agent postoperatively. Treatment guidelines in the immediate post-splenectomy phase need to be formulated to avoid thrombotic complications. Multiple agents have been shown to be of benefit; however, use of aspirin and or low molecular weight heparin should be suggested in the absence of absolute contraindications. As in our case, fear of bleeding prevents more ubiquitous use of pharmacologic prophylaxis in these patients. Other agents such as melphalan, hydroxyurea and plateletpheresis remain good options especially in cases where symptomatic extreme reactive thrombocytosis develops; but given the lack of prospective studies it may be difficult to advise for or against one particular approach.

## Abbreviations

ACCP: American College of Chest Physicians
AIHA: Autoimmune hemolytic anemia
BMI: Body mass index
CMS: Centers for Medicaid and Medicare Services
DVT: Deep vein thrombosis
EKG: Electrocardiogram
ES: Evan’s Syndrome
ET: Essential thrombocythemia
ITP: Immune thrombocytopenia
IVIG: Intravenous immunoglobulin
LDH: Lactate dehydrogenase
MCV: Mean corpuscular volume
MPN: Myeloproliferative neoplasm
PE: Pulmonary embolism
PMP: Platelet microparticles
PSVT: Portal or splenic vein thrombosis
RNA: Ribonucleic acid
RP: Reticulated platelets
RP%: Reticulated platelet percentage
RT: Reactive thrombocytosis
SCDs: Sequential compression devices
VTE: Venous thromboembolism
w-AIHA: Warm autoimmune hemolytic anemia
